# Revealing seed color variation and their possible association with yield and quality traits in a diversity panel of flax (*Linum Usitatissimum* L.)

**DOI:** 10.3389/fpls.2022.1038079

**Published:** 2022-11-11

**Authors:** Mozhgan Abtahi, Aghafakhr Mirlohi, Negar Sharif-Moghaddam, Ehsan Ataii

**Affiliations:** Department of Agronomy and Plant Breeding, College of Agriculture, Isfahan University of Technology, Isfahan, Iran

**Keywords:** chromatic parameters, CIELAB color space, diversity panel, flax, luminosity parameter, quality traits

## Abstract

Seed color is a vital quality determinant of flax, significant for consumers’ acceptability, and determines the commercial values of seeds. Also, seed color as a phenotypic marker may be a convenient way to select the plants with desired traits. This study assessed a diversity panel representing 144 flax genotypes from diverse geographical origins for the existence of genetic variability for luminosity (L*) and chromaticity (a* and b*) seed color parameters, seed yield, and quality traits over two years. The genetic variance was significant for seed color parameters, demonstrating the presence of significant genetic variability, which provides a resource to objectively evaluate and select flax genotypes based on seed color according to the market demand. High heritability combined with the high genotypic coefficient of variation observed for seed yield, oil, and protein content suggested a better genetic gain upon selecting these traits. Seed yield, seed quality traits, and phenological traits showed significant negative correlation with L* and b* parameters and positive correlation with a* suggesting that the seeds’ dark background and brown color can serve as marker characters to prescreen early-flowering, high-yielding and oil and protein-rich genotypes. Interestingly 48 brown-seeded genotypes were identified as early-flowering with short height, large seeds, high thousand seed weight, and capsule diameter. In addition, 34 genotypes were characterized by light-colored yellow seeds, large seeds, late-flowering with shorter height, and high branch numbers. Our results highlighted that North America and Australia-belonged genotypes were lighter yellow-seeded than the ones from other continents. Flax genotypes from South America and Asia were high-yielding, while genotypes from North America were low-yielding genotypes. Moreover, darker brown-seeded genotypes have prevailed in the South American continent.

## Introduction

Using flaxseed (*Linum Usitatissimum* L.) is advantageous, owing to its healthy nutritional patterns, long back cultivation history, and functional properties (Bangar et al., 2021). Flaxseed has been the focus of growing interest for nutritionists and medical researchers due to its promising biological activities viz; exceptionally high content of alpha-linolenic acid (ALA), flavonoids, phytoestrogens including lignans, and high quality protein (Deluca et al., 2018; Bangar et al., 2021). Flax seeds exhibit wide phenotypic diversity in the size, shape, and color of seeds (Nozkova et al., 2014). Seed size and seed coat color have been used to develop a convenient method for seed quality improvement in several crop species, including soybean (Marwanto, 2004), rapeseed (Zhang et al., 2008), flax (Dana and Ivo, 2008), and common bean (Possobom et al., 2015). Seed color is an important characteristic of flax associated with the marketability of flax cultivars. Seed quality and nutritional value of seeds are affected mainly by the amount, composition, and polymerization degree of tannins in the outer seed coats (Bangar et al., 2021; Tomaszewska-Gras et al., 2021). The natural color of mature flax seeds varies from deep brown to light yellow through different intermediates. Yellow flaxseeds have thin and soft hulls, and result from blocked biosynthesis of proanthocyanidins (condensed tannins) that impart a brown color to the seed coat (Kajla et al., 2015; Xu et al., 2015). Based on the linolenic acid content of the seeds, yellow-seeded flax are of two types named ‘Omega’ and ‘Solin’. Brown-seeded flax and yellow-seeded flax variety ‘Omega’ both contain more than 50 percent alpha-linolenic acid in the oil, are susceptible to oxidative deterioration, and are unsuitable for food and cooking purposes. To bypass the high oxidation instability, the low linolenic-yellow seeded varieties with the trademark of ‘Solin’ containing less than five percent linolenic fatty acid in seed oil have been developed for edible purposes using mutation breeding (Sudarshan et al., 2017). In addition to its direct influence on the acceptability of flax varieties and nutritional quality, seed coat color in flax is linked with essential breeding traits and biochemistry of protein and oil metabolism (Sudarshan, 2013; Lindenback, 2015; Zare et al., 2021). It is important to understand these associations because these traits may be genetically linked. The genes involved in seed coat color may even be pleiotropic, means that a single gene affects the expression of multiple phenotypic traits. By understanding the relationships between seed coat color and other traits, flax breeders can use seed coat color as a phenotypic marker, or they can avoid undesirable characteristics when selecting seed color (Lindenback, 2015; Sudarshan et al., 2017).

Seed color determination may be visually assessed by a rating color scale or quantified by instrumental analysis (Pathare et al., 2013). The colorimeter method (CIE LAB system) allowed the determination of the seed coat color of flax genotypes with high-accuracy for luminosity values (L*) and chromaticity attributes (a* and b*). L* value has been used by breeders to select genotypes regarding the clarity of the seed coat. Chromaticity a* and b* values (specify the shades in pairs of colors) have been used to determine the differences in the seed color of flax varieties (Hu et al., 2020; Tomaszewska-Gras et al., 2021). This happens because flax seeds of different types may have similar L* values, but vary by the chromaticity a* and b* values. Therefore, chromaticity a* and b* values, along with L* value, express the intensity of the seed color in more detail.

Despite the longstanding interest in flax seed color, genetic control and inheritance of flax seed coat color using L*, a*, and b* parameters are still poorly understood. Considering that the seed color is a central target in flax breeding programs, the present investigation was planned to unravel the genetics of seed coat color parameters and their possible associations with important traits and characterize a large panel of flax genotypes with diverse origin based on seed color, agronomic and quality traits.

## Materials and methods

### Plant material and experimental design

The 144 flax genotypes used in this study were selected from a genetically diverse population containing oilseed type genotypes mainly from IPK world collection (Gatersleben, Germany), eight Iranian genotypes, three Canadian breeding lines, a commercial cultivar (Flanders), and two genotypes from India (**Table 1**). The geographical distribution of the genotypes included Asia, Africa, Australia, Europe, North America, Central America, and South America. Field evaluation of genotypes was done in a randomized complete block design with two replications and over two years (2016 and 2017) at the Research Farm of the Isfahan University of Technology (51° 28' E Long., 32° 42' N Lat., 1624 m a.s.l.) with annual average temperature of 17°C and annual precipitation of 122 mm with no summer rain. In each replication, seeds of each genotype were planted in two rows, each 1.5 meters long with 25 cm inter-row spacing and a distance of 1 cm between the plants within the row. Each row contained 150 plants of each genotype. Spacing between two replicates was 2 meters.

**Table 1:** Information on flax genotypes used in this study.

Genotype number	Accession name	Country of origin	Genotype number	Accession name	Country of origin	Genotype number	Accession name	Country of origin
1	IRI16	Iran	49	USA1579	United States	97	ISR1393	Israel
2	GRC1102	Greece	50	CHL1171	Chile	98	DEU729	Germany
3	CAN 2022	Canada	51	MARS52	Morocco	99	DZA1835	Algeria
4	BEL1829	Belgium	52	DNK897	Denmark	100	SUN1096	Russian
5	IRIe25	Canada	53	PRT121	Portugal	101	ARG1155	Argentina
6	SLV588	El Salvador	54	HUN1585	Hungary	102	GTM633	Guatemala
7	FRA771	France	55	IRI78	Iran	103	CAN899	Canada
8	CAN790	Canada	56	DEU1011	Germany	104	CHN175	China
9	TUR1238	Turkey	57	URY165	Uruguay	105	indian	India
10	CSK380	Czechoslovakia	58	YUG230	Iran	106	AFG1127	Afghanistan
11	HUN762	Hungary	59	CAN358	Canada	107	IND214	Iran
12	COL1771	Colombia	60	CSK1110	Czechoslovakia	108	IRL761	Canada
13	ESP122	Spain	61	AUT186	Austria	109	IRL1867	Ireland
14	SUN1440	Russian	62	DEU717	Germany	110	GTM1863	Guatemala
15	HUN778	Hungary	63	DEU814	Germany	111	BLR877	Belarus
16	SUN1404	Russian	64	IRIb1	Iran	112	AUT1019	Austria
17	SWE715	Sweden	65	POL1027	Poland	113	NZL1563	New zealand
18	DEU2096	Germany	66	ETH238	Ethiopia	114	ESP1192	Spain
19	ISR867	Israel	67	GTM1459	Guatemala	115	ESP1192	Non
20	ARG794	Argentina	68	ARG825	Argentina	116	ERI 208	Eritrea
21	HUN1571	Hungary	69	MAR168	Morocco	117	NPL1794	Iran
22	TUR1251	Turkey	70	DEU702	Germany	118	AF1133	Afghanistan
23	IRI1213	Iran	71	BEL1828	Belgium	119	AUT1086	Austria
24	PRK1779	North korea	72	RUS1866	Russian	120	FRA848	France
25	IRIb66	Iran	73	IRIm1	Iran	121	ARG1869	Argentina
26	DEU1010	Germany	74	TWN1821	Taiwan	122	CHN1201	China
27	ISR1008	Israel	75	FRA806	France	123	ARG2139	Argentina
28	IRN89	Iran	76	ETH811	Ethiopia	124	IRIe33	Canada
29	AUT1200	Austria	77	ETH811	Ethiopia	125	CHN1561	Canada
30	GRC1275	Greece	78	MAR1047	Morocco	126	BGR123	Bulgaria
31	JPN1820	Japan	79	SWE1429	Iran	127	HUN601	Iran
32	MAR1029	Morocco	80	ROM1075	Romania	128	AUS626	Australia
33	PRT423	Portugal	81	ETH231	Ethiopia	129	AUT948	Austria
34	CAN 2154	Canada	82	AFG1147	Afghanistan	130	CYP1199	Cyprus
35	IRIe33	Canada	83	URY1182	Uruguay	131	CYP1199	Cyprus
36	AUS590	Australia	84	ARG864	Argentina	132	PRT1099	Iran
37	DEU956	Germany	85	sp1066	Canada	133	GEO1780	Georgia
38	USA1578	United States	86	YEM202	Yemen	134	LTU1474	Lithuania
39	IND1343	India	87	BEL1115	Belgium	135	IRIKH77	Iran
40	BRA1227	Brazil	88	AFG402	Afghanistan	136	ARG772	Argentina
41	LTU952	Lithuania	89	ETH312	Ethiopia	137	DDR2146	Germany
42	CAN1362	Iran	90	IRQ1375	Iraq	138	ARG2091	Argentina
43	FIN1446	Finland	91	USA1570	United States	139	ITA1792	Italy
44	CYP706	Cyprus	92	HUN259	Hungary	140	ITA1791	Italy
45	CHE1050	Switzerland	93	CHE1051	Switzerland	141	DNK2094	Denmark
46	USA1580	United States	94	IRI960	Iran	142	AUT1186	Austria
47	CHN718	China	95	ko37	Iran	143	CHN857	China
48	LVA752	Latvia	96	FRA770	France	144	FRA1167	France

### Trait measurements

Thirteen agro-morphological traits were recorded on 20 randomly selected plants from each genotype in each replicate during two years. Days to germination (DG), days to flowering (DF), and days to capsule formation (DC) were recorded as the number of days from sowing until 50% of plants started germination, flowering, and capsule formation, respectively. Days to maturity (DM) was recorded as the period from sowing until 50% brown capsules, i.e. when seeds rattled in the capsules. Plant height (PH) was measured at the maturity stage from the ground to the most upper part of the plant. Number of branches per plant (NB) was recorded as the number of lateral shoots on the main stem. Number of capsules per plant (NC) was determined by counting the total number of bolls on the plant. Capsule diameter (CD) was determined as the average diameter of 50 random capsules using a caliper. Number of seeds per Capsule (NSC) was recorded as the average seed number in 50 random capsules. Thousand seed weight (TSW) was measured by scaling 1000 harvested seeds using a sensitive balance. Plots are harvested soon after physiological maturity, seed yield (SY) was recorded in grams per square meters and then converted into kg per hectare. Also, SY per plant was considered the average weight of total harvested seeds on 20 randomly selected plants. Seed length (SL) and seed width (SW) were recorded as the average length and width of 50 random seeds. Then, seed quality traits including oil content (OIL), and protein content (PRO) were determined by near-infrared (NIR) spectroscopy analyzer model DA 7250 monochromator instrument (Perten Instruments, Hagersten, Sweden) with three replications.

### Seed coat color determination

The seeds harvested from each flax genotype were stored at room temperature, and air-dried, clean, and undamaged seeds were used for color determination. To accurately determine the parameters describing color, and understand color variation, diffuse reflectance spectroscopy at a spectrum of wavelengths from 400 to 740 nm were recorded on UV-Vis spectrophotometer UV4 (Unicam, UK) using Labsphere holder (Labsphere Inc., USA) and Vision 3.0 software (Unicam, UK). The white spectral standards were used for measuring the background on both spectrometers. Each sample was measured five times. Flaxseed color was determined by calculating three parameters (L*, a*, and b*) based on the International Commission on Illumination (CIE) color solid scale using Microsoft Excel 2010 software (Cui et al., 2021; Tomaszewska-Gras et al., 2021).

### Multivariate statistics

Diffuse reflectance Vis (% R) spectra in ASCII format and normalized values of colorimetric parameters (L*, a*, and b*) were exported to Statistica 12.0 (Statsoft, USA) software for multivariate statistical analysis. Combined analysis of variance and estimation of variance components were performed using PROC Mixed in SAS 9.2 (SAS Institute, 2002). Statistically significant differences among genotypes were determined by the LSD test at P  =  5%.

The phenotypic and genotypic coefficient of variation (PCV and GCV) was calculated using the following equation from Falconer and Mackay (1996):


(1) PCV = (σp/µ)× 100

(2) GCV = (σg/µ)× 100

Where σp and σg are the standard deviation of the phenotypic and genotypic effect, respectively and µ is the phenotypic mean. Broad-sense heritability was calculated using the ratio of Hallauer and Miranda (1988):


(3) $$h^{2}{\;}_{b}\left(for\;combined\;years\right)\;=\;\sigma^{2}g/\left(\sigma^{2}g\;+\;\sigma^{2}gy/y\;+\;\sigma^{2}gr/r\;+\sigma^{2}e/ry\right)$$

In which σ^2^_g_ is the genotype variance, σ^2^_gy_ is the genotype×year variance, σ^2^_gr_ is the genotype×replication variance, σ^2^_e_ is the environmental variance due to genotype×replication×year, and r and y are the number of replicates and years, respectively.

Principal component analysis (PCA; covariation matrix) of the data were made and graphical outputs, i.e. dendrograms of similarity and component score graphs were created using JMP software. Phenotypic correlation among traits was calculated using Stat Graphics software ver. 17.2 (Statgraphics, 2016). The R (v2.5, http://cran.r-project.org/) package “ggplot2” was used to draw boxplots (Alvarado et al., 2020).

## Results

### Genetic variation of agronomic and seed quality traits

The results of ANOVA revealed highly significant variations for agro-morphological traits and seed quality characters among the 144 flax genotypes (**Table 2**). The effect of year and the interaction of genotype and year were significant for most measured traits. There was a marked genetic difference for seed yield (SY) ranging from 140 kg/ha to 1250 kg/ha, showing broad genetic variability among the genotypes. Oil content (OIL) ranged from 20.04% to 39.60%, and protein content (PRO) from 28.84% to 48.14% (**Table 3**). An extensive variation was observed for most measured traits, with the genotypic coefficient of variation ranging from 4.45% to 43.16% and the phenotypic coefficient of variation from 5.13% to 52.50%. SY, OIL, and PRO had the most considerable genetic variation (**Table 3**). Based on the mean performance, seven genotypes (86, 117, 95, 127, 136, 50, and 132) were the best-performing ones, with a mean SY of 640 kg/ha, compared to others with an average yield of 580 kg/ha (**Table S1**). Regarding OIL, six genotypes (132, 57, 136, 105, 17, and 112) were superior with an average of 35% compared with the others having an average of 30% (**Table S1**). Concerning PRO, six genotypes (3, 73, 8, 20, 57, and 19) with an average of 46%, were superior to other genotypes, compared to the overall average of 40% (**Table S1**). Seed quality traits, including OIL and PRO, had relatively high heritability values (H^2^ = 0.69 and 0.73, respectively) (**Table 3**). Highest heritability estimates were obtained for seed length (SL) and seed width (SW) (H^2^ = 0.93). Except for SY (H^2^ = 0.51), heritability estimates were relatively high (ranging from 0.64 to 0.78) for most agro-morphological traits (**Table 3**).

**Table 2:** Combined ANOVA for measured traits in 144 flax genotypes evaluated during two years.

Characters	Year (Y)(df= 1)	Replication/Y(df= 2)	Genotype (G)(df= 143)	G× Y(df= 143)	Error(df= 286)
DG	17896.49**	15.22	19.18^**^	18.80^**^	12.62
DF	10609^**^	20.67	53.69^**^	13.13^**^	6.33
DC	22717.18^**^	3.47	31.67^**^	17.11^**^	8.84
DM	11247.78^**^	34.33	25.54^**^	8.30^**^	5.15
PH	554.34^**^	265.12	1.74^**^	0.87^**^	0.65
NB	218.71^**^	8.88	1038.03^**^	3.08^**^	73.95
NC	29355.11^**^	3349.00	184.70^**^	159.56^*^	108.20
NSC	1.29^NS^	14.98	1.56^**^	0.40^NS^	0.46
CD	0.06^NS^	1.37	0.32^**^	0.57^NS^	0.05
TSW	51.53^**^	1.94	1.57^**^	0.41^**^	0.21
SY	11895.23^**^	941.70	602.27^**^	506.51^**^	0.56
SL	0.59^**^	0.09	0.36^**^	0.02^**^	0.01
SW	0.54^**^	0.02	0.08^**^	0.006^NS^	0.005
L*	586.04^**^	33.15	122.40^**^	58.26^*^	7.63
a*	145.97^**^	7.56	62.00^**^	6.17^NS^	5.53
b*	1321.80^*^	346.52	466.00^**^	313.99^NS^	310.77
a/b	0.63^**^	0.01	0.08^**^	0.01^NS^	0.007
OIL	187.31^**^	18.96	16.54^**^	6.96^**^	1.71
PRO	96.75^**^	18.12	19.52^**^	12.58^**^	2.77

NS not significant, *p< 0.05 and **p< 0.01.

DG days to germination, DF days to flowering, DC days to capsule formation, DM days to maturity, PH plant height, NB number of branches, NC number of capsule, NSC number of seed per capsule, CD capsule diameter, TSW thousand seed weight, SY seed yield, SL seed length, SW seed width, L*, a*, b*, and a/b color parameters, OIL oil content, PRO protein content.

**Table 3:** Estimates of genetic parameters and phenotypic performance of 19 traits evaluated in 144 flax genotypes during two years.

Trait	Mean± SD	Range	PCV (%)			GCV(%)	H^2^b
DG	23.87 ± 3.25	14-35		8.44	7.72		0.78
DF	69.76 ± 7.25	60-87		7.65	6.61		0.76
DC	79.18 ± 9.31	67-102		5.13	4.45		0.76
DM	97.82 ± 6.75	90-116		7.04	5.81		0.75
PH (cm)	52.48 ± 5.90	24.2-91		41.76	35.69		0.77
NB	5.85 ± 0.32	4-11		37.50	31.88		0.64
NC	25.98 ± 4.87	14-95.20		35.68	30.73		0.65
NSC	8.41 ± 0.90	4.80-9.80		40.54	34.34		0.72
CD (mm)	6.40 ± 0.35	4.87-7.67		37.43	31.27		0.70
TSW (g)	3.76 ± 0.87	1.53-8.21		43.99	31.97		0.66
SY (kg/ha)	580.83 ± 10.13	140.05-1250.69		52.50	43.16		0.51
SL (mm)	4.17 ± 0.29	3.45-5.18		17.83	17.03		0.93
SW (mm)	2.20 ± 0.16	1.84-2.65		16.67	16.43		0.93
L*	51.24 ± 5.13	39-68		25.38	24.40		0.91
a*	22.63 ± 1.49	9-31.25		27.35	26.47		0.88
b*	36.37 ± 2.78	29.25-47.75		33.78	31.93		0.89
a/b	0.64 ± 0.05	0.20-1.03		33.44	32.09		0.90
OIL (%)	30.50 ± 1.75	20.40-39.60		46.12	41.55		0.69
PRO (%)	40.21 ± 2.12	28.84-48.14		45.91	40.32		0.73

DG days to germination, DF days to flowering, DC days to capsule formation, DM days to maturity, PH plant height, NB number of branches, NC number of capsule, NSC number of seed per capsule, CD capsule diameter, TSW thousand seed weight, SY seed yield, SL seed length, SW seed width, L*, a*, b*, and a/b color parameters, OIL oil content, PRO protein content.

### Genetic variation of seed coat color parameters

The effects of genotype, year, and interaction effects for the lightness/darkness (L*), redness/greenness (a*), yellowness/blueness (b*), and redness/yellowness ratios (a/b) are shown in **Table 2**. The effects of genotype and year were significant for all color values. The genotype × year interaction was significant for the L* parameter, suggesting that it varied for the flax genotypes in both years (**Table 2**). However, the variation of L* due to the year appears minimal because the ranges were almost the same across the years (data not shown).

**Figure 1:**
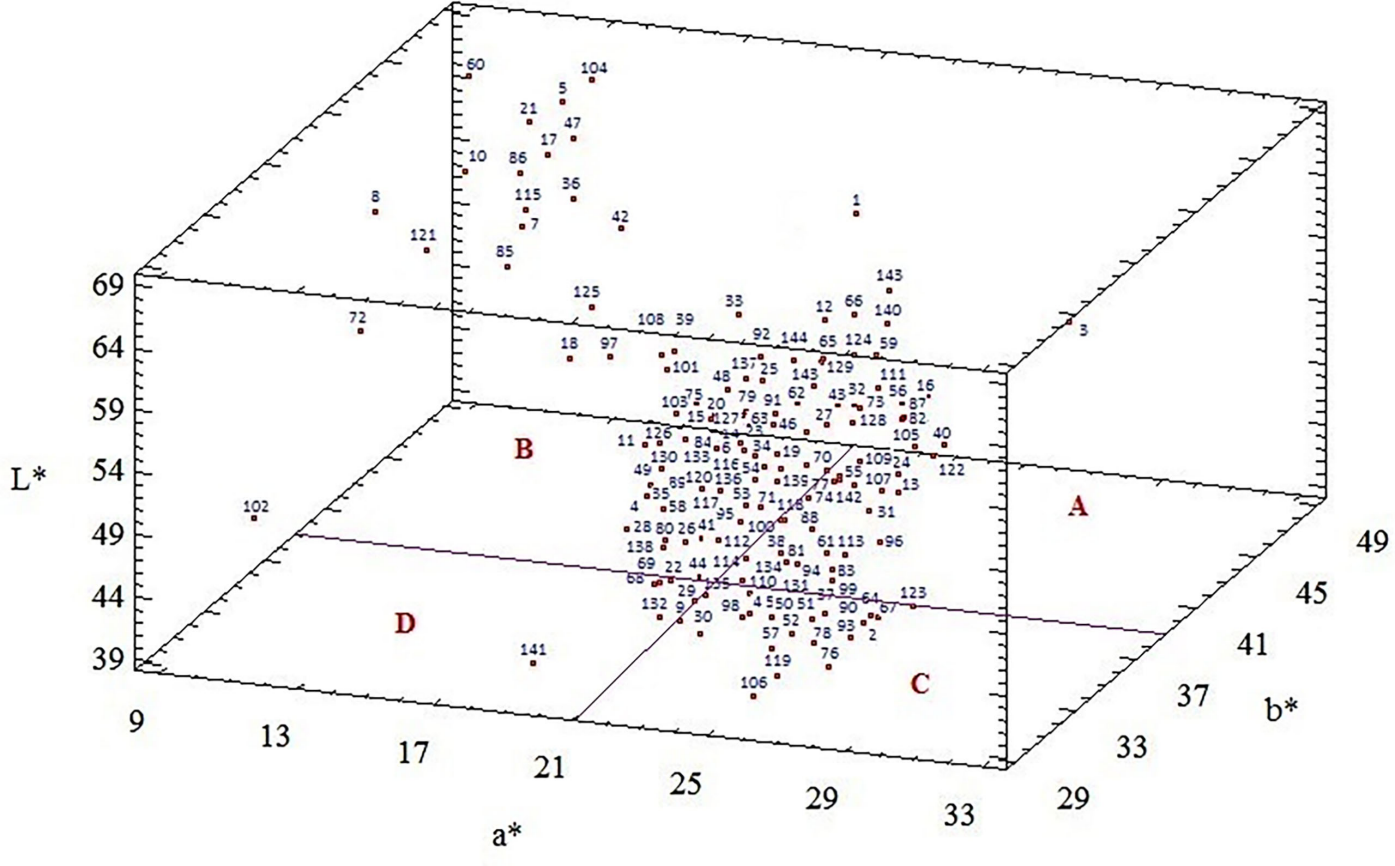
CIE L*, a*, b* color diagram for seeds of 144 flax genotypes. Part **(A)** consisted of genotypes with intermediate to high values of color parameters a*, b*, and L*. Part **(B)** included genotypes with low to medium values of a* parameter and intermediate to high values of b* and L* parameters. Part **(C)** contained genotypes with intermediate to high values of a* and low to medium values of the b* and L*. Part **(D)** included genotypes with low to medium values of a*, b*, and L* parameters.

An average L*, a*, b*, and a/b values of 144 flax genotypes were 51.24, 22.63, 36.37, and 0.64, respectively (**Table 3**). The CIE L*, a*, and b* color diagram for 144 flax genotypes is presented in **Figure 1**. Flax genotypes showed wide variations in seed coat color parameters. The L*-value exhibited a wide range of 39-69. This parameter was significantly lower for genotypes 141, 46, 76, 49, 51, 134,102, 64, and 58 (from 39.5 to 46) and higher for genotypes 60, 21, 36, 47, 5, 42, 115, and 8 (from 63.5 to 69) (**Table S1**). The values of a*, b*, and a/b parameters were in the range of 9-33, 29-49, and 0.06-1.03, respectively. The seed coat color of genotypes 60, 8, 72, 121, 21, and 36 had the lowest a* values (from 9 to 12), while genotypes 46, 3, 106, 118, 131, 76, 113, and 119 registered the highest values (from 27 to 33). The b* values were the lowest (from 29 to 32) for genotypes 141, 134, 46, 64, 50, 112, and 105 and highest (from 45 to 49) for genotypes 104, 5, 21, 125, 47, 36, and 60 (**Table S1**). The genotypes 60, 8, 21, 121, 36, 72, 47, 5, and 10 provided the lowest values of a/b ratios (0.2-0.29), while genotypes 46, 106, 119, 113, 134, 76, 105, and 118 had the highest values (from 0.81-1.03) (**Table S1**). Five genotypes (1, 3, 4, 143, and 128) showed intermediate to high values of color parameters a*, b*, and L*. Twenty-one genotypes (5, 7, 8, 10, 17, 18, 21, 42, 43, 47, 60, 36, 72, 85, 86, 97, 104, 115, 121, 128, and 125) registered low to medium values of a* parameter and intermediate to high values of b* and L* parameters. Three genotypes (48, 102, and 141) provided low to intermediate values of color parameters a*, b*, and L*. Seven genotypes (25, 33, 59, 65, 66, 79, and 144) expressed intermediate to high values of parameters a* and low to medium values of the b* and L* parameters. Finally, 108 genotypes registered intermediate to high values of the a* parameter and low to intermediate values of b* and L* (**Table S1**). The evident difference in these parameters was found between genotypes 141 and 102 and genotypes 1, 3, and 143; the cluster corresponding to former genotypes is located in the D quadrant due to the lowest values of color parameters L*, a*, and b*, while the cluster corresponding to genotypes 1, 3, and 143 is located in the A quadrant due to the highest values of these parameters (**Figure 1**).

The genotypic coefficient of variation ranged between 24.40%-32.09% and phenotypic coefficient of variation ranged between 25.38%-33.78% (**Table 3**). High heritability estimates were observed for the L* value (H^2^ = 0.91), a* (H^2^ = 0.88), b* (H^2^ = 0.89), and a/b ratio (H^2^ = 0.90) (**Table 3**).

### Association among seed coat color parameters, seed yield and seed quality traits

Based on genotypic correlations between character pairs, SY exhibited the strongest positive association with number of seeds per capsule (NSC; 0.79), SW; 0.73, capsule diameter (CD; 0.69), number of capsules (NC; 0.68), thousand seed weight (TSW; 0.66), number of branch (NB; 0.65), and SL; 0.63. Also, SY was significantly correlated with OIL (0.60) and PRO (0.62) (**Table 4**). Statistically negative correlations were determined between the SY and color parameters L* (-0.70) and b* (-0.59), and positive correlations were determined between the SY and color parameter a* (0.67), and a/b ratio (0.58) (**Table 4**). Interestingly oil content was positively associated with SL (0.73), SW (0.69), TSW (0.66), CD (0.58), PRO (0.56), a* (0.55), and a/b ratio (0.54). Furthermore, negative associations were determined between the oil content and L* (-0.56) and b* (-0.53) parameters (**Table 4**). Protein content was positively correlated with SL (0.57), SW (0.55), a* parameter (0.57), and a/b ratio (0.51) (**Table 4**). On the other hand, a positive coefficient of correlation was obtained between TSW, SW, and SL with a* parameter (0.60, 0.64, and 0.59, respectively). A significant negative correlation was found between days to flowering (DF) and the a* parameter (-0.57), a/b ratio (-0.55), CD (-0.55), NSC (-0.56), SL (-0.53), SW (-0.51), OIL (-0.56), PRO (-0.59), and SY (-0.63) and significant positive correlation between DF and L* (0.59), and b* (0.58) parameters (**Table 4**). Significantly high positive correlation coefficients were found between L* and b* values (0.90), and negative correlations were found between L* and a* (-0.88) values, L* value and a/b ratio (-0.91). Chromatic parameters a* and b* were negatively associated (-0.74) (**Table 4**).

**Table 4:** Genotypic correlation coefficient used to assess correlation among the seed coat color, seed yield parameters and quality traits of 144 flax genotypes.

Traits	DG	DF	PH	NB	NC	NSC	CD	TSW	SY	SL	SW	L*	a*	b*	a/b	OIL	PRO
DG	1																
DF	0.37*	1															
PH (cm)	-0.38*	0.32*	1														
NB	-0.36*	-0.57**	-0.32*	1													
NC	-0.34*	0.06	0.03	0.41**	1												
NSC	-0.39**	-0.56**	-0.11	-0.30*	-0.11	1											
CD (mm)	0.16-	-0.55**	-0.33*	0.32*	0.35*	-0.10	1										
TSW (g)	0.02	-0.38*	-0.48**	0.33*	0.12	-0.07	0.64**	1									
SY (kg/ha)	-0.36*	-0.63**	-0.60**	0.65**	0.68**	0.79**	0.69**	0.66**	1								
SL (mm)	0.03	0.53**-	-0.42**	0.36*	0.15	-0.38**	0.71**	0.80**	0.63**	1							
SW (mm)	0.04	-0.51**	-0.38**	0.38**	0.07	0.70**	0.78**	0.78**	0.73**	0.87**	1						
L*	-0.09	0.59**	0.11	0.03	0.06	-0.35**	-0.06	-0.22	-0.70**	0.16	0.17	1					
a*	0.04	-0.57*	0.02	0.02	-0.07	0.35**	0.05	0.60**	0.67**	0.59**	0.64**	-0.88**	1				
b*	-0.11	0.58**	0.07	0.07	0.03	-0.38**	-0.10	-0.34*	-0.59**	0.10	0.009	0.90**	-0.74**	1			
a/b	0.10	-0.55**	-0.03	-0.03	-0.06	0.38**	0.10	0.38**	0.58**	-0.22	-0.04	-0.91**	0.93**	-0.88**	1		
OIL (%)	0.04	-0.56**	-0.21	0.04	0.21	0.14	0.58**	0.66**	0.60**	0.73**	0.69**	-0.56**	0.55**	-0.53**	0.54**	1	
PRO (%)	-0.05	-0.59**	-0.19	-0.18	0.03	0.16	0.16	0.05	0.62**	0.57**	0.55**	-0.54**	0.57**	-0.56**	0.51**	0.56**	1

*p< 0.05 and **p< 0.01.

DG days to germination, DF days to flowering, DC days to capsule formation, DM days to maturity, PH plant height, NB number of branches, NC number of capsule, NSC number of seed per capsule, CD capsule diameter, TSW thousand seed weight, SY seed yield, SL seed length, SW seed width, L*, a*, b*, and a/b color parameters, OIL oil content, PRO protein content.

### Discrimination of flax genotypes based on biplot analysis

**Figure 2:**
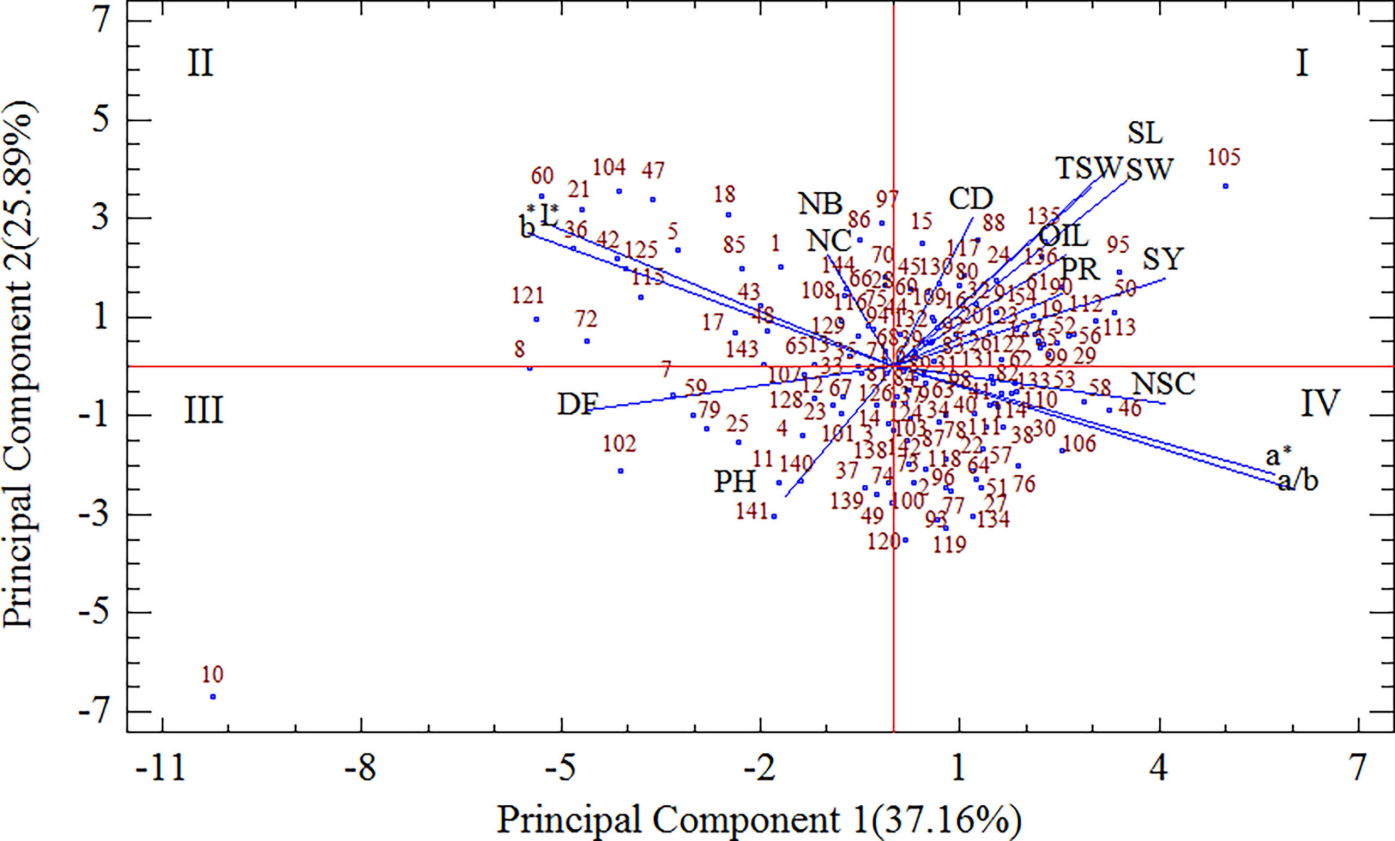
The biplot depicting the scatter of 144 flax genotypes alongside the measured traits. DF days to flowering, PH plant height, NB number of branches, NC number of capsule, NSC number of seed per capsule, CD capsule diameter, TSW thousand seed weight, SY seed yield, SL seed length, SW seed width, L*, a*, b*, and a/b color parameters, OIL oil content, PRO protein content.

Principal component analysis (PCA) was carried out to assess the variation of agro-morphological traits, quality characters, color parameters, and profile differences among flax genotypes. The first principal component (PC1), which explained 37.16% of the variance, was positively correlated with the a* parameter and a/b ratio, and negatively correlated with L* and b* parameters and DF. The second principal component (PC2) accounted for 25.89% of total variance and was positively attributed to SL, SW, TSW, and CD, and negatively related to PH. **Figure 2** depicts the plot of loadings, which visualizes relations between variables by analyzing the first two PCs. Furthermore, 144 genotypes were placed in 4 different quartiles of biplot based on the positive or negative signs of the sectors of the PC1 and PC2 (**Figure 2**). The group “I” was formed by 48 genotypes on the positive side of both PC1 and PC2 and densely scattered close to the SY, OIL, PRO, and CD. Group “II” included 34 genotypes on the negative side of PC1 and positive side of PC2 and distributed around the NC, NB, L* and b* color parameters. Twenty-three genotypes on the negative side of both PC1 and PC2 were more diverse around the PH and DF and were grouped in region “III”. It is noteworthy that there was an evident distinction of genotype 10 with the lowest values of both PCs from other genotypes. Finally, 39 genotypes distributed in the positive side of PC1 and negative side of PC2 and close to the NSC, and grouped in region IV of the biplot (**Figure 2**).

### Diversity pattern within and between geographical origins

**Figure 3:**
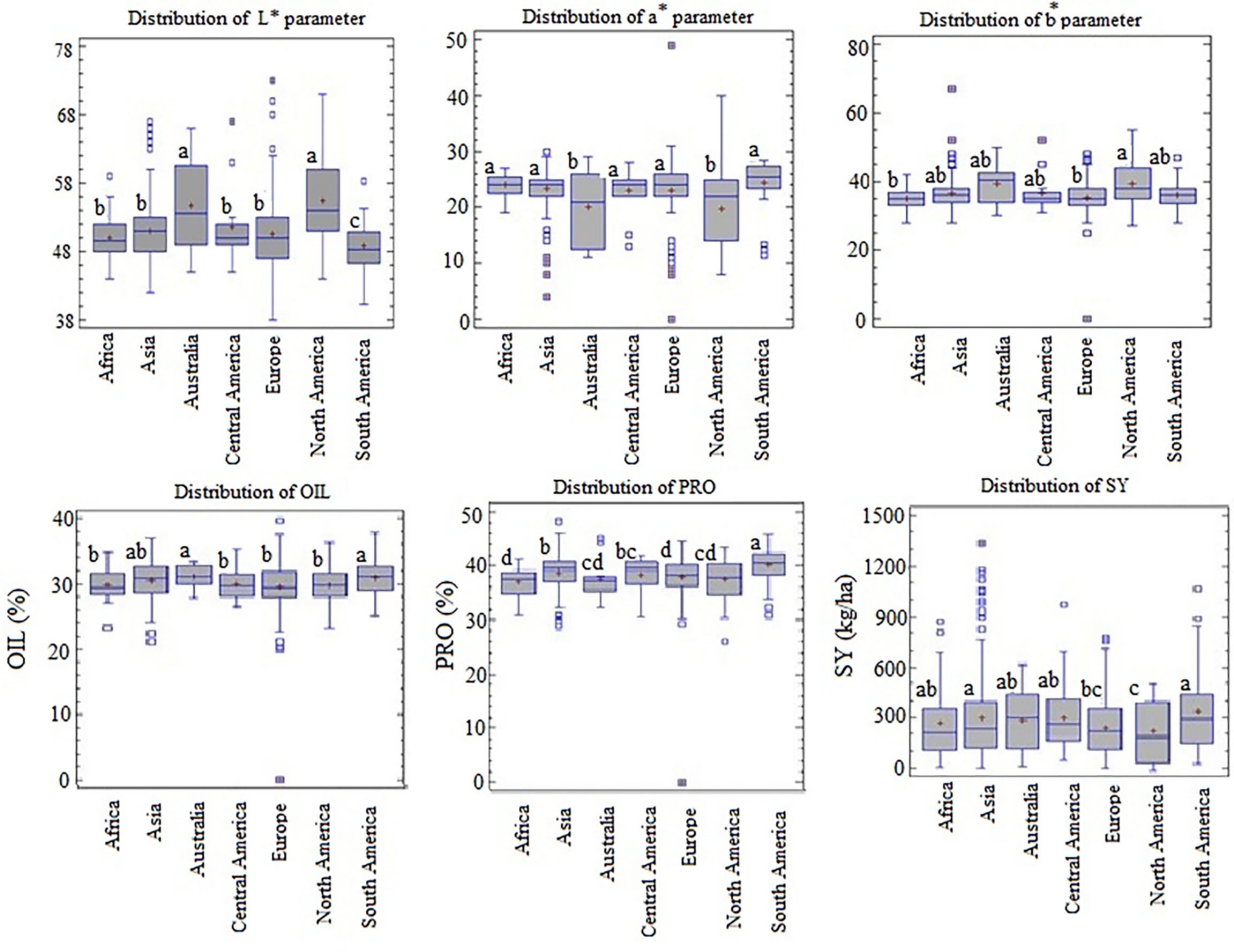
Variation distribution of seed color parameters, quality traits and seed yield of 144 flax genotypes from 7 different geographical origins over two years. The different letters in the upper plot denote statistically significant differences among the geographic origin (LSD = 0.05).

The variation in seed color parameters, seed yield, oil, and protein content among the seven continental regions (Asia, Australia, Europe, North America, Central America, South America, and Africa) is shown in **Figure 3**. Box edges denote the upper and lower quartile, with the median value shown as a bold line in the center of the box. Whiskers represent 1.5 times the interquartile range of the data. Individual points beyond the whiskers are shown as open dots. The boxplots obtained from different continents for each trait were compared statistically, and the LSD test determined the significant differences among means of the geographical origins at P = 5% in **Figure 3**. Accordingly, significant differences were observed for PRO at least between three continents. These differences were considerably high between the two continents of South America and Africa, with the highest and lowest PRO content, respectively. Significant differences for SY were observed at least between three continents. The most significant statistical difference was detected between South America and North America, with the highest and lowest SY, respectively. For OIL, significant differences were observed between at least two of the continents. The oil content of Australian and South American genotypes was significantly higher than that of Africa, Central America, Europe, and North America. For the L* parameter, at least two continents showed a significant difference. The mean values of the L* parameter were slightly higher in genotypes originating from North America and Australia, whereas the lowest mean values were observed for South America genotypes. Significant differences were observed between at least two continents for the b parameter. The values of the b* parameter were considerably high for North America and low for the African and Europe continents. For the a* parameter, significant differences were observed between at least two continents. The mean performance of the a* parameter was significantly high for South America and low for Australia and North America.

## Discussion

The analysis of variance showed highly significant differences among the 144 flax genotypes for agro-morphological and seed quality traits which is encouraging for genotypic selection. The significant genetic variability observed in the present study relates to germplasm collection size from very diverse geographical origins. Significant variance due to year and the genotype × year interaction for most measured traits, suggested that the relative performance of the genotypes changed across the two experimental years and different genotypes responded to environmental conditions differentially. High estimates of genotypic coefficient of variation for SY, OIL, and PRO content can meet the flax breeding objective in evolving a high-yielding and oil and protein-rich cultivar (Roy and Shil, 2020). Relatively high heritability values, ranging from 0.64 to 0.93 for agro-morphological and seed quality traits, indicated that genetic variance predominates in total phenotypic variance and there is ample scope for improving these traits through selection (Saroj et al., 2021). This finding is in line with other studies that reported high heritability values for agro-morphological and seed quality traits in flax (You et al., 2017; Dabalo et al., 2020; Walkowiak et al., 2022).

**Figure 4:**
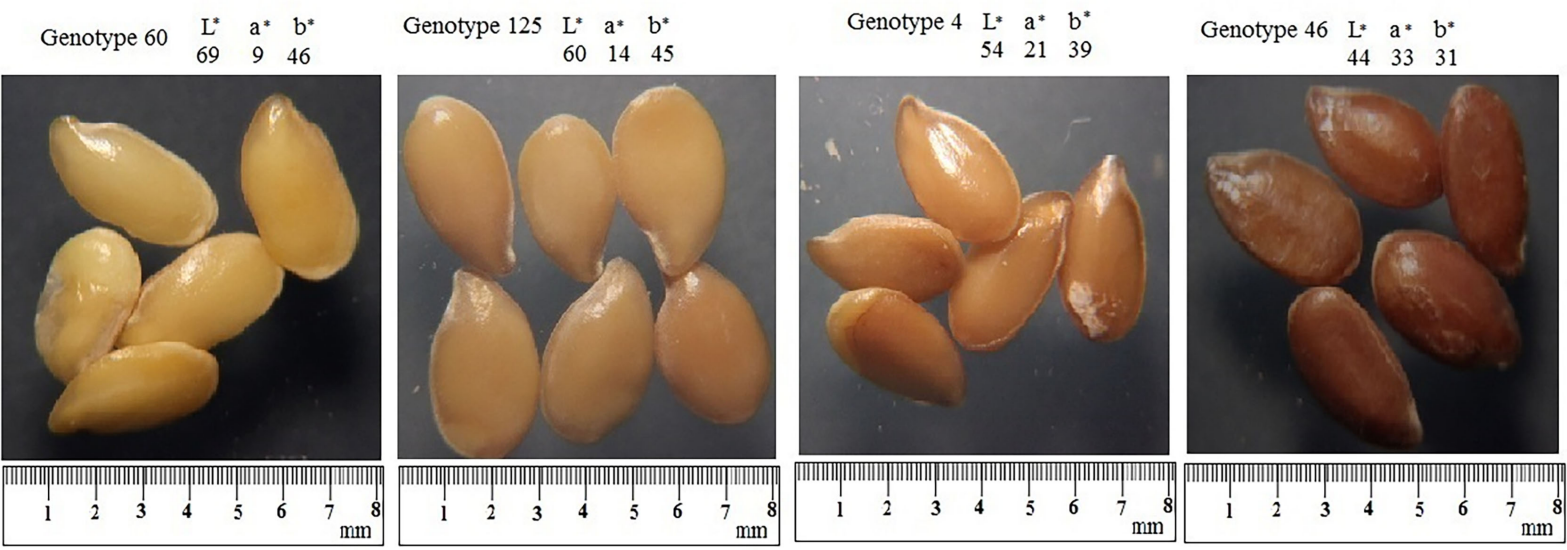
Four seed color types in the flax collection used and their L*, a*, and b* values.

Seed color is an important quality determinant for consumers’ acceptability (Tomaszewska-Gras et al., 2021); therefore, calculations of three parameters describing color were performed. Considerable variability was also observed among the flax genotypes for seed color parameters, as shown by the range of variation for L*, a*, and b* parameters originating from the variation in the amount of seed pigments (García-Fernández et al, 2021; Gebregziabher et al., 2022). Thus, selection for luminosity and chromaticity (a* and b*) values in flax seeds will be effective, meeting the demand of a diverse market niche. The L* value represents lightness and clarity ranging from darkest black (0) to brightest white (100), a* evaluates the variation of the green to red shades (red = positive value and green = negative value), and the b* measures the variation of the blue to yellow shades (yellow = positive value and blue = negative value) (Cui et al., 2021). Therefore, in the studied germplasm, it is possible to select flax genotypes with darker or dull (lower L*-value) and transparenter or shine (higher L*-value) seeds and reddish-brown (higher a*-value) and yellow seeds (higher b*-value) (**Figure 4**). Considering the selection for lower L* values, below the average of 144 flax genotypes, it was possible to identify 114 genotypes with less transparency and lightness. It was also possible to select flax genotypes with higher clarity of seed coat as 21 genotypes exceeded the established limits of L* value from the mean value observed in the 144 flax genotypes. Given that all values of a* and b* parameters were positive in the studied germplasm, the trend mainly included reddish-brown and yellowish shades, respectively. The lightness and yellowness were most frequent for seeds of 21 genotypes showing low values of color parameter a* and high values of L* and b*. In comparison, the darkness and redness were more pronounced in 108 genotypes registering high values of color parameter a* and low values for L* and b*. Five genotypes (1, 3, 4, 143, and 128) were found to have the highest levels of yellowness, redness, and lightness. Among the 144 flax genotypes, three genotypes (48, 102, and 141) showed the lowest values of the three color parameters. Usually, genotypes with higher L* values and the highest chromaticity a* and b* values will present a more transparent seed coat background and secondary reddish-brown or yellow color (Possobom et al., 2015). It is known that the pigmentation of the seed coat color is mainly determined by tannins (proanthocyanidins). It was reported that dark coat or brown genotypes of flaxseeds have a higher concentration of anthocyanins and proanthocyanidins than lighter color or yellow ones, offering a valuable source of antioxidants. In contrast, light coat or yellow flaxseeds contain no tannins, but the yellow cotyledons are rich in carotenoids (Troshchynska et al., 2019).

In terms of the seed coat color parameters, heritability estimates from 0.88 to 0.91 infer ease in selecting the flax genotypes based on the three parameters (Possobom et al., 2015). Similarly, other researchers verified high heritability for the color values in sesame and bean (Possobom et al., 2015; Cui et al., 2021; Sadohara et al., 2021). For flaxseed, broad and narrow-sense heritability estimates are not reported for luminosity, chromaticity a* and b* values.

Seed coat color is a central target in flax, and deleterious traits correlated to it maybe avoided when selecting for color in a breeding programs (Sudarshan et al., 2017; Zare et al., 2021). The negative correlations between seed yield and L* and b* parameters suggest that seed yield decreases as the lightness and yellowness of the seed increase in flax. On the other hand, positive correlations between seed yield and a* parameter and a/b ratio indicated that seed yield increases as the redness or brownness, and redness/yellowness ratio of seed increases. Other studies in flax showed that yellow-seeded genotypes had lower seed yield which was attributed to decreased seed vigor, poorer stands, lower germination rate, and the absence of flavonoids in the yellow seeds (Saeidi and Rowland, 1999; Lindenback, 2015; Zare et al., 2021). In addition, a negative correlation was observed between both quality traits (OIL and PRO) and the color parameters L* and b*. In effect, the yellow-seeded genotypes contain, on average less OIL and PRO than brown-seeded ones. A high positive coefficient of correlation between the OIL and PRO and a* parameter suggests that the red or brown color of the seeds may be used efficiently for indirect selection of oil and protein-rich genotypes. Similarly, reddish-brown seed coats genotypes of *Brassica rapa* showed higher oil content than that of yellow seeded genotypes (Bagheri et al., 2012). Also, results showed that selection based on big capsules and heavy-weight seeds could be a valid approach for developing genotypes rich in oil which is also in line with the findings of Siddiqui et al. (2016) and Ataii et al. (2021). A significant correlation between TSW, SW, and SL with a* parameter suggested that larger seeds often had a brown color. Positive correlations between DF with L* and b* parameters represent that late-flowering genotypes usually have lighter yellow seeds, and as seed color become darker, they mature faster. These results were coherent with other studies indicating that flax genotypes with yellow seed coats were late maturing (Lindenback, 2015; Tavarini et al., 2016; Sudarshan et al., 2017). Also, DF had a significant negative correlation with OIL, PRO, and SY, suggesting that early flowering would favorably increase seed yield, oil, and protein content since it allows access to nutrients for seed filling, resulting into superior seed yield (Siddiqui et al., 2016). Taken together, genotypes with a brown seed coat color may be preferentially selected to develop early-flowering, high-yielding, high oil and protein-rich cultivars. Positive correlation estimates between L* and b* color parameters and negative estimates between L* and a*, and a* and b* indicates that darker background genotypes (dull coat color) have higher a* and lower b* values. Although L* parameter is able to distinguish shiny seed coats from dull, but is limited by color influence (parameters a* and b*). This highlights the complexity of the seed color trait and that it would be inappropriate to independently select for the color parameters during seed color breeding programs (Black and Panozzo, 2004; Possobom et al., 2015). Therefore, chromaticity a* and b* values, along with luminosity L* value, express the intensity of the seed coat color in more detail.

PCA led to satisfactory discrimination of the 144 flax genotypes. The similarity of the genotypes was evaluated using principal component analysis, and four groups were suggested. The sign of PC1 separated the flax genotypes according to seed color: negative for light yellow-seeded genotypes and positive for darker brown-seeded forms. In addition, the genotypes were well separated from each other by the sign of PC2 according to yield components: negative for PH and positive for SL, SW, TSW, and CD. Genotypes in region I were early-flowering short stem with large reddish-brown seeds. Among them, genotype 105 registered the highest values of a* parameter, and the lowest values of PH and DF. Genotypes in region II were late-flowering with shorter stems and larger yellow seeds with high NC and NB. Among them, genotypes 60, 21, 104, 36, and 47 registered the highest values of L* and b* parameters, and the lowest values of PH. Genotypes in region III were late-flowering with longer stems and smaller yellow seeds with low TSW, and CD. It was noteworthy that genotype 10 showed the highest values of L* and b* parameters, DF, and PH and the lowest values of SL, SW, TSW, and CD. Genotypes occupied regions IV were early-flowering with longer stems and smaller reddish-brown seeds with low TSW, and CD.

Geographical distribution of flax genotypes indicated the existence of wide variability for seed color parameters, yield and quality traits, attributed to genetic factors and growing conditions such as humidity, temperature, rainfall, and shorter exposure to sunlight (Sadohara et al., 2021; Gebregziabher et al., 2022). Significant differences among continents for seed color parameters indicated that their grown seeds had different colors. The L* value of the North America and Australia genotypes appeared to be considerably higher, indicating that genotypes from these continents had lighter seeds than other continents. Whereas the lowest and highest mean values of L* and a* parameters, respectively were observed in genotypes originated from Africa, Asia, Central America, Europe, and South America, indicating that genotypes from these continents were dark brown-seeded types with high amounts of tannins. The higher b* value of the North American genotypes indicated a more intense yellow color and hence the presence of increased amounts of carotenoids. Indeed, genotypes from North America showed high values of the L* parameter and low values of a* parameter, SY, and OIL. In contrast, genotypes from South America had low values of the L* parameter and high values of a* parameter, SY, OIL, and PRO. These results interestingly confirm our previous findings suggesting that darkness and brownness of seed coat may be used as important parameters to prescreen high-yielding genotypes of flax with high oil and protein content.

## Conclusion

The present study revealed that flax genotypes with wide geographic distribution and considerable genetic variation for agronomic traits, followed by quality attributes and seed color parameters which bids significant opportunities for future breeding programs. Thus, from this germplasm, selecting flax genotypes based on seeds color according to the market demand with dark or transparent background and brown or yellow seed color was possible. The results suggested that indirect selection for increased flax yield is possible by selecting for higher seed weight and brown seed color. Significantly superior oil and protein content was found in brown flax seeds, confirming the importance of the brown seed color for these two seed quality traits. Forty eight genotypes were identified as early-flowering genotypes with short height and large brown seeds with high thousand seed weight and capsule diameter. Moreover, 34 genotypes were characterized as late-flowering with shorter height, light-colored yellow seeds, large seeds, and high branch numbers. North America’s genotypes were lighter yellow with lower seed yield and oil content than the ones from other continents. Dark brown-seeded genotypes predominate in Africa, Asia, Central America, Europe, and South America, while yellow-seeded ones mainly belong to North America and Australia. Our results also highlighted those darker brown seeded genotypes with the highest seed yield, oil, and protein content prevail in the South American continent.

## Data availability statement

The raw data supporting the conclusions of this article will be made available by the authors, without undue reservation.

## Author contributions

MA was responsible for extracting and genetic data analysis, interpreting and discuss the results and drafting the manuscript, writing original draft preparation. AM conceived and supervised the project and contributed to draft the manuscript, NS-M participated in the project and contributed to data extraction, EA participated in the project and assisted in the data collection. All authors contributed to the article and approved the submitted version.

## Funding

This work is based upon research funded by Iran National Science Foundation (INSF) under project No. 4004449.

## Conflict of interest

The authors declare that the research was conducted in the absence of any commercial or financial relationships that could be construed as a potential conflict of interest.

## Publisher’s note

All claims expressed in this article are solely those of the authors and do not necessarily represent those of their affiliated organizations, or those of the publisher, the editors and the reviewers. Any product that may be evaluated in this article, or claim that may be made by its manufacturer, is not guaranteed or endorsed by the publisher.
