# Supplementary material for: Revealing seed color variation and their possible association with yield and quality traits in a diversity panel of flax (Linum Usitatissimum L.)
Source: Front Plant Sci. 2022 Nov 11;13:1038079. doi: 10.3389/fpls.2022.1038079 (PMC9691844; doi:10.3389/fpls.2022.1038079)
Supplement: Supplementary file 1 [file Table_1.docx]

| **Table S1.** Average values of 19 traits evaluated in 144 flax genotypes during two years. | | | | | | | | | | | | | | | | | | |
| --- | --- | --- | --- | --- | --- | --- | --- | --- | --- | --- | --- | --- | --- | --- | --- | --- | --- | --- |
| Genotype number | DF | DC | DM | PH (cm) | NB | NC | CD (mm) | NSC | TSW (g) | SY (kg/ha) | SL (mm) | SW (mm) | L* | a* | b* | a/b | OIL(%) | PRO(%) |
| 1 | 73.5 | 83.75 | 97.75 | 56.45 | 3.1 | 60.8 | 6.586833 | 9.066667 | 3.339995 | 513.1225 | 4.6585 | 2.4045 | 58 | 23.5 | 42.25 | 0.556213 | 28.85 | 40.02 |
| 2 | 68.5 | 77 | 96 | 64.35 | 1.9 | 27.9 | 6.040833 | 9.016667 | 3.047325 | 522.3733 | 4.46 | 2.2995 | 49.5 | 24.75 | 33.75 | 0.733333 | 24.75 | 38.61 |
| 3 | 72 | 80 | 96 | 75.075 | 1.35 | 15.55 | 6.294833 | 8.666667 | 3.416925 | 518.1833 | 4.6785 | 2.38125 | 55.5 | 29 | 40 | 0.725 | 27.25 | 43.69344 |
| 4 | 73.5 | 78.5 | 96 | 67.525 | 1.2 | 16.15 | 6.081167 | 8.616667 | 3.182361 | 520.0108 | 4.57125 | 2.28925 | 54.5 | 21.75 | 38.5 | 0.564935 | 26.475 | 39.90079 |
| 5 | 70.5 | 78.5 | 97.75 | 49.775 | 1 | 28.25 | 6.3675 | 8.616667 | 3.491623 | 521.0473 | 4.59725 | 2.433 | 63.75 | 13.75 | 47.25 | 0.291005 | 30.825 | 41.29205 |
| 6 | 70 | 77 | 97.75 | 58.25 | 1.05 | 27.8 | 6.378833 | 9.083333 | 3.726433 | 528.8738 | 4.59875 | 2.4025 | 51.5 | 22.5 | 36.75 | 0.612245 | 29.875 | 41.07521 |
| 7 | 71 | 78.5 | 96 | 61.9 | 1 | 21.9 | 6.041667 | 8.133333 | 2.299835 | 311.1758 | 4.59725 | 2.32325 | 58.75 | 13.5 | 38.25 | 0.352941 | 27.86532 | 35.78917 |
| 8 | 72.75 | 80 | 96 | 69 | 1.1 | 20 | 5.865833 | 6.966667 | 2.746639 | 311.651 | 4.4285 | 2.2405 | 63.5 | 9.75 | 44 | 0.221591 | 28.9 | 42.44083 |
| 9 | 73.75 | 82 | 98.75 | 51.575 | 1.6 | 15.1 | 6.345833 | 8.7 | 3.229144 | 534.2595 | 4.654 | 2.3745 | 52 | 23 | 35.75 | 0.643357 | 30.675 | 40.02281 |
| 10 | 80.5 | 91.5 | 100.5 | 57.3 | 2.05 | 22.75 | 6.673 | 6.633333 | 3.570649 | 303.3188 | 2.29 | 1.13125 | 57.5 | 13 | 44 | 0.295455 | 26.11 | 37.34 |
| 11 | 77 | 82 | 96.75 | 60.05 | 1.4 | 20.7 | 5.858833 | 8.2 | 2.228573 | 519.3615 | 4.417 | 2.28175 | 52.75 | 22.5 | 36.5 | 0.616438 | 31.125 | 37.07917 |
| 12 | 75.5 | 84 | 97.75 | 41.975 | 1.3 | 18.5 | 6.125833 | 8.65 | 3.101733 | 314.683 | 4.56425 | 2.38425 | 53.75 | 22.75 | 37.25 | 0.610738 | 27.25 | 34.8425 |
| 13 | 68.25 | 77 | 96 | 46.475 | 2 | 20.95 | 6.472833 | 6.5 | 3.384196 | 519.7605 | 4.535 | 2.4065 | 51.75 | 24 | 37.75 | 0.635762 | 28.65 | 38.00095 |
| 14 | 72 | 77 | 96 | 56.375 | 1.7 | 23.8 | 6.307833 | 8.55 | 3.231431 | 522.1975 | 4.616 | 2.34475 | 50.25 | 22.75 | 35.25 | 0.64539 | 29.4 | 40.24284 |
| 15 | 70.5 | 82 | 97.5 | 47.15 | 1.7 | 21.3 | 6.855167 | 6.766667 | 4.810714 | 517.8663 | 5.005 | 2.54525 | 52 | 22.5 | 36.75 | 0.612245 | 32.225 | 39.68539 |
| 16 | 72.75 | 78.5 | 96.75 | 56.425 | 2.5 | 35.6 | 6.420833 | 8.183333 | 3.846979 | 529.0833 | 4.81475 | 2.45075 | 50.75 | 24.75 | 36 | 0.6875 | 32.725 | 40.50292 |
| 17 | 71 | 77 | 96 | 60.25 | 0.7 | 26.45 | 6.132167 | 8.583333 | 3.226343 | 530.7563 | 4.55525 | 2.36775 | 58 | 12.75 | 39.25 | 0.324841 | 33.65 | 39.81311 |
| 18 | 72.75 | 78.5 | 96.75 | 59.3 | 1.85 | 27.05 | 6.826333 | 8.366667 | 4.36158 | 526.2715 | 4.73025 | 2.45175 | 60.25 | 13.5 | 42.75 | 0.315789 | 31.55 | 38.79279 |
| 19 | 68.75 | 77 | 97.75 | 55.575 | 2 | 21.4 | 6.910333 | 8.016667 | 4.087468 | 532.1125 | 4.8655 | 2.44825 | 48.5 | 25.5 | 32.5 | 0.784615 | 31.875 | 41.64964 |
| 20 | 67.5 | 77 | 96.75 | 40.7 | 1.55 | 19 | 6.337167 | 8.183333 | 3.909986 | 444.7378 | 4.62475 | 2.42525 | 50.5 | 23 | 36.25 | 0.634483 | 30.7 | 42.12201 |
| 21 | 73.75 | 82 | 98.75 | 53.35 | 2.3 | 23 | 6.389667 | 7.216667 | 3.756446 | 523.8233 | 4.64275 | 2.441 | 67.5 | 11.5 | 47.25 | 0.243386 | 29.15 | 36.56855 |
| 22 | 68.25 | 78.5 | 97.75 | 47.8 | 1.8 | 32.15 | 6.1285 | 8.116667 | 3.562506 | 533.0188 | 4.4345 | 2.28525 | 47 | 25.75 | 32.25 | 0.79845 | 31.3325 | 39.82794 |
| 23 | 72 | 78.5 | 97.75 | 61.525 | 1.7 | 31.75 | 6.477167 | 8.766667 | 3.26654 | 518.1288 | 4.498 | 2.332 | 52 | 22 | 36.75 | 0.598639 | 27.67358 | 36.02083 |
| 24 | 65.5 | 77 | 96.75 | 56.325 | 1.45 | 30.65 | 6.814667 | 8.816667 | 4.431865 | 536.3573 | 4.81075 | 2.475 | 52.25 | 23.75 | 37.25 | 0.637584 | 33.425 | 37.96069 |
| 25 | 77.75 | 85.75 | 104.75 | 43.375 | 2.15 | 9.75 | 6.068125 | 7.508333 | 3.565888 | 509.8698 | 4.7555 | 2.4315 | 51.25 | 23.75 | 38.75 | 0.612903 | 30.11 | 35.1 |
| 26 | 69.25 | 82 | 96.75 | 44.025 | 1.9 | 21.1 | 6.351333 | 8.466667 | 3.730437 | 530.9768 | 4.76425 | 2.47975 | 50 | 22.25 | 35.25 | 0.631206 | 30.475 | 36.85144 |
| 27 | 66.5 | 78.5 | 96 | 62.175 | 1.15 | 19.75 | 6.043167 | 8.466667 | 2.93041 | 526.739 | 4.42375 | 2.34675 | 48 | 26.25 | 32.75 | 0.801527 | 30.9 | 38.94381 |
| 28 | 73.75 | 82 | 101.25 | 46.275 | 1.95 | 32.65 | 6.282167 | 7.916667 | 3.990732 | 533.725 | 4.87075 | 2.44725 | 53.75 | 22 | 37.5 | 0.586667 | 32.9 | 40.76453 |
| 29 | 64.5 | 77 | 96 | 53.6 | 1.45 | 23.65 | 6.508333 | 8.3 | 4.017054 | 542.013 | 4.71925 | 2.46425 | 49 | 24.25 | 33.25 | 0.729323 | 30.525 | 40.87705 |
| 30 | 67.5 | 78.5 | 96.75 | 44 | 2.1 | 22.25 | 6.442 | 8.283333 | 3.328098 | 534.5648 | 4.6405 | 2.38125 | 47.25 | 26.25 | 33.5 | 0.783582 | 29.25 | 36.88848 |
| 31 | 67.75 | 77 | 97.75 | 46.125 | 1.35 | 19.4 | 6.375833 | 8.45 | 3.318119 | 533.578 | 4.55025 | 2.33525 | 52.5 | 23.5 | 36.25 | 0.648276 | 29.425 | 39.78787 |
| 32 | 68.75 | 78.5 | 96.75 | 48.35 | 1.35 | 19.8 | 6.753 | 9.066667 | 4.103706 | 534.998 | 4.8025 | 2.47075 | 53.5 | 23 | 36.5 | 0.630137 | 31.325 | 37.4222 |
| 33 | 74.5 | 84 | 98.5 | 48.8 | 1.75 | 23.2 | 6.509 | 8.683333 | 3.572669 | 522.3435 | 4.655 | 2.3975 | 51.75 | 23.25 | 38.75 | 0.6 | 17.45 | 41.26801 |
| 34 | 72 | 80 | 98.5 | 59.65 | 2.05 | 27.15 | 6.286167 | 8.883333 | 3.353253 | 519.2588 | 4.6145 | 2.36825 | 50.25 | 25.25 | 33.5 | 0.753731 | 27.425 | 40.15 |
| 35 | 72.75 | 78.5 | 98.5 | 58.975 | 1.7 | 30.95 | 6.379167 | 9 | 3.387472 | 512.8715 | 4.68275 | 2.38525 | 52.5 | 23 | 38 | 0.605263 | 29.61757 | 38.85417 |
| 36 | 73.75 | 82 | 97.5 | 59.7 | 2.7 | 31.35 | 6.3415 | 7.366667 | 3.483066 | 514.76 | 4.67075 | 2.29525 | 65 | 12 | 45.75 | 0.262295 | 29.835 | 39.9 |
| 37 | 72 | 78.5 | 96 | 56.45 | 1.6 | 16.6 | 6.205 | 8.05 | 2.800953 | 515.3275 | 4.372 | 2.28475 | 47.5 | 24.25 | 35 | 0.692857 | 26.53 | 33.87831 |
| 38 | 68.75 | 80 | 96.75 | 58.675 | 0.1 | 17.65 | 6.275833 | 8.3 | 3.49995 | 541.2648 | 4.64775 | 2.40525 | 48.25 | 26.5 | 35.5 | 0.746479 | 32.025 | 37.45674 |
| 39 | 67.5 | 80 | 96.75 | 49.075 | 2.15 | 19.4 | 6.255667 | 8.033333 | 2.749342 | 533.664 | 4.786 | 2.461 | 51 | 22.5 | 37 | 0.608108 | 32.725 | 37.89562 |
| 40 | 69 | 78.5 | 96.75 | 55.4 | 2.2 | 22.9 | 6.277 | 8.266667 | 3.673374 | 539.919 | 4.54575 | 2.35325 | 48 | 26 | 33.75 | 0.77037 | 29.9 | 38.60148 |
| 41 | 68.5 | 77 | 96.75 | 59.05 | 1.95 | 26.7 | 6.616833 | 8.683333 | 3.665475 | 538.6878 | 4.64525 | 2.32875 | 48.5 | 25.25 | 33 | 0.765152 | 28.35 | 39.02951 |
| **Table S1.** Continued. | | | | | | | | | | | | | | | | | | |
| Genotype number | DF | DC | DM | PH (cm) | NB | NC | CD (mm) | NSC | TSW (g) | SY (kg/ha) | SL (mm) | SW (mm) | L* | a* | b* | a/b | OIL(%) | PRO(%) |
| 42 | 72.75 | 80 | 100.25 | 46.9 | 2.2 | 41.55 | 6.4625 | 7.333333 | 3.064832 | 515.223 | 4.4835 | 2.34725 | 63.75 | 13.25 | 42.5 | 0.311765 | 30.85 | 38.12494 |
| 43 | 77.25 | 84 | 101.25 | 40.6 | 2.5 | 12.95 | 6.1315 | 8.466667 | 3.593898 | 521.9703 | 4.634 | 2.412 | 54 | 19.25 | 44.5 | 0.432584 | 32.3 | 41.49743 |
| 44 | 67.5 | 77 | 97.75 | 53.9 | 1.45 | 32.35 | 6.384667 | 7.15 | 3.955162 | 520.118 | 4.74825 | 2.46775 | 49.5 | 23.5 | 35 | 0.671429 | 28.725 | 37.76366 |
| 45 | 72 | 80.5 | 97.5 | 37 | 2.25 | 36.25 | 7.668278 | 7.15 | 4.635816 | 506.8835 | 4.941 | 2.62875 | 46.25 | 23.25 | 32.5 | 0.715385 | 25.11 | 34.1 |
| 46 | 66.5 | 77 | 96.75 | 48.75 | 1.9 | 23.15 | 6.372167 | 7.266667 | 3.319568 | 531.616 | 4.8805 | 2.49775 | 44.25 | 33.25 | 32 | 1.039063 | 30.4 | 37.8475 |
| 47 | 71.25 | 80 | 98.5 | 59.275 | 2.4 | 24.25 | 6.686667 | 7.383333 | 3.941663 | 532.488 | 4.68525 | 2.4605 | 64.5 | 12.5 | 46 | 0.271739 | 30.35 | 35.96751 |
| 48 | 75.25 | 85.75 | 98.5 | 48.325 | 3.85 | 33.25 | 6.334844 | 7.963333 | 3.283656 | 309.9198 | 4.5935 | 2.38225 | 53 | 20.25 | 37.5 | 0.54 | 24.875 | 37.7775 |
| 49 | 72 | 78.5 | 96 | 62.775 | 0.45 | 24.75 | 6.245167 | 7.753111 | 2.850416 | 412.6048 | 4.4595 | 2.322 | 45.5 | 23 | 32.5 | 0.707692 | 16.4 | 41.11068 |
| 50 | 66.5 | 77 | 97.5 | 51.925 | 1.85 | 25.75 | 6.6633 | 9.296874 | 4.729505 | 652.12 | 4.82225 | 2.468 | 46.75 | 23.25 | 31.25 | 0.744 | 32.1775 | 39.9359 |
| 51 | 67.5 | 77 | 96.75 | 59.275 | 1.35 | 24.85 | 6.043576 | 9.137569 | 2.993591 | 538.316 | 4.35825 | 2.3215 | 45.75 | 25.5 | 34.75 | 0.733813 | 27.25 | 35.92112 |
| 52 | 70.5 | 78.5 | 97.5 | 51.725 | 1.15 | 25.65 | 6.203272 | 8.820282 | 4.634317 | 548.468 | 4.80025 | 2.47275 | 48.5 | 25.25 | 33.75 | 0.748148 | 32.65 | 39.66972 |
| 53 | 65.5 | 78.5 | 98.5 | 45.15 | 2.1 | 26.35 | 6.20694 | 8.498019 | 3.828501 | 546.5078 | 4.77525 | 2.4395 | 48.75 | 23.5 | 32 | 0.734375 | 31.775 | 38.74822 |
| 54 | 67.5 | 78.5 | 97.5 | 46.9 | 2.35 | 23.1 | 6.609352 | 8.603201 | 4.022011 | 525.2 | 4.77525 | 2.48775 | 47.5 | 23.75 | 32.75 | 0.725191 | 32.2 | 37.92547 |
| 55 | 67.5 | 77 | 97.75 | 50.975 | 1.5 | 23.3 | 6.675357 | 8.73688 | 4.211137 | 533.5955 | 4.851 | 2.462 | 48.25 | 24.25 | 33.75 | 0.718519 | 30.575 | 39.49169 |
| 56 | 66.25 | 77 | 96.75 | 49.875 | 1.2 | 30.7 | 6.670757 | 9.084681 | 4.144535 | 542.4408 | 4.71275 | 2.476 | 47.75 | 25.5 | 34.25 | 0.744526 | 29.55 | 40.81815 |
| 57 | 66.5 | 77 | 96 | 54.875 | 1 | 22 | 6.254606 | 8.238979 | 3.262458 | 522.402 | 4.44325 | 2.33175 | 47.25 | 24.5 | 32.25 | 0.75969 | 34.9 | 41.78006 |
| 58 | 65.5 | 77 | 96 | 37.25 | 0.95 | 16.4 | 6.365174 | 9.001487 | 3.83749 | 536.5448 | 4.57625 | 2.4025 | 46 | 26 | 32.75 | 0.793893 | 31.425 | 39.8575 |
| 59 | 80.25 | 91.5 | 104.75 | 40.425 | 4.25 | 18.1 | 5.866089 | 7.961766 | 3.332972 | 512.673 | 4.71275 | 2.3905 | 52.25 | 22.75 | 39 | 0.583333 | 30.11 | 37.32 |
| 60 | 73 | 80 | 96.75 | 45.025 | 2 | 40.15 | 6.657167 | 7.166667 | 3.186767 | 517.7598 | 4.5215 | 2.36525 | 69 | 9 | 45 | 0.2 | 29.82074 | 39.5325 |
| 61 | 66.5 | 77 | 96 | 60.95 | 0.9 | 23.3 | 6.7665 | 8.866667 | 4.668233 | 533.8073 | 4.86025 | 2.491 | 50.25 | 24.75 | 36 | 0.6875 | 30.975 | 41.02819 |
| 62 | 69.75 | 77 | 97.5 | 52.125 | 2.75 | 26.15 | 6.4505 | 8.9 | 3.48212 | 541.3013 | 4.77 | 2.41425 | 48.75 | 24.5 | 33.25 | 0.736842 | 31.525 | 33.05617 |
| 63 | 72.75 | 82 | 98.5 | 49.15 | 2.8 | 24.6 | 6.657667 | 8.366667 | 3.164192 | 521.9395 | 4.5875 | 2.44475 | 49 | 25 | 35.25 | 0.70922 | 24.025 | 35.87828 |
| 64 | 72.75 | 80.5 | 98.75 | 71.25 | 2 | 24.5 | 6.082333 | 8.916667 | 3.456982 | 518.0205 | 4.64375 | 2.39625 | 46 | 25.25 | 31.25 | 0.808 | 27.9025 | 37.19333 |
| 65 | 77.75 | 87.75 | 106.5 | 50.3 | 3.35 | 21.8 | 6.245833 | 8.283333 | 3.081389 | 420.7295 | 4.63225 | 2.363 | 52.25 | 23.75 | 39 | 0.608974 | 29.725 | 40.13923 |
| 66 | 72 | 82.25 | 98.5 | 51.425 | 2.9 | 30.15 | 6.862833 | 8.816667 | 3.267557 | 512.1025 | 4.72175 | 2.496 | 52.75 | 22.75 | 40.75 | 0.558282 | 29.45 | 34.79412 |
| 67 | 72.75 | 80 | 98.5 | 55.45 | 2.6 | 23.15 | 6.2005 | 8.083333 | 3.30687 | 512.0355 | 4.65725 | 2.37175 | 49.75 | 24.25 | 35.25 | 0.687943 | 29.025 | 35.08206 |
| 68 | 72.75 | 80 | 98.5 | 55.1 | 2.95 | 22.35 | 6.4595 | 8.65 | 3.327151 | 521.9685 | 4.68675 | 2.41425 | 50.75 | 22.75 | 36.25 | 0.627586 | 29.46923 | 35.6625 |
| 69 | 67.5 | 80.75 | 98.5 | 57.4 | 1.75 | 35.4 | 6.626167 | 7.383333 | 3.908762 | 533.9593 | 4.81075 | 2.50175 | 51 | 22.75 | 36.75 | 0.619048 | 30.025 | 40.59116 |
| 70 | 72 | 78.5 | 97.5 | 47.825 | 3.05 | 32 | 6.769833 | 8.033333 | 4.217916 | 509.268 | 4.82525 | 2.51625 | 52.5 | 22 | 35.5 | 0.619718 | 27.15 | 33.4975 |
| 71 | 72 | 83.75 | 97.75 | 49.125 | 1.975 | 44.525 | 6.592958 | 7.770833 | 3.388651 | 514.352 | 4.6515 | 2.41925 | 49 | 22.5 | 33.75 | 0.666667 | 27.825 | 34.76917 |
| 72 | 72 | 77 | 97 | 60.9 | 1.45 | 25.65 | 5.996167 | 6.266667 | 3.08548 | 512.1053 | 4.52475 | 2.3165 | 59.25 | 10.5 | 39 | 0.269231 | 29.29513 | 34.59417 |
| 73 | 68.25 | 77 | 96 | 69.775 | 1.8 | 21.1 | 6.232333 | 8.383333 | 3.251546 | 422.1898 | 4.5115 | 2.342 | 49 | 25 | 35 | 0.714286 | 15.75 | 42.85594 |
| 74 | 72 | 80 | 96.75 | 75.225 | 1.6 | 22.15 | 6.386 | 9.133333 | 2.933308 | 514.4675 | 4.37675 | 2.35475 | 48.75 | 24.75 | 35.5 | 0.697183 | 26.25 | 37.95417 |
| 75 | 77 | 84 | 106.5 | 44.025 | 3.55 | 34.6 | 6.196667 | 9.216667 | 3.793329 | 540.438 | 4.501 | 2.379 | 51 | 23 | 36 | 0.638889 | 28.925 | 39.87885 |
| 76 | 66.5 | 77 | 96 | 58.55 | 1.4 | 31.5 | 6.130833 | 8.9 | 3.140443 | 531.0498 | 4.41675 | 2.302 | 45.5 | 27.25 | 33.25 | 0.819549 | 33.225 | 41.3611 |
| 77 | 68.5 | 77 | 96 | 60.825 | 0.75 | 21.85 | 6.0555 | 9.133333 | 3.041972 | 520.2143 | 4.461 | 2.28575 | 48 | 24.25 | 33.75 | 0.718519 | 29.525 | 39.06746 |
| 78 | 67.5 | 80 | 97.5 | 34.475 | 1.85 | 19 | 6.024667 | 8.772222 | 3.23381 | 332.6625 | 4.5095 | 2.3215 | 48.5 | 25.75 | 35.75 | 0.72028 | 28.975 | 37.56511 |
| 79 | 82 | 91.5 | 106.5 | 42.275 | 2.85 | 15.25 | 6.082958 | 7.383333 | 4.135751 | 512.1125 | 4.351 | 2.1945 | 52.25 | 23 | 38.75 | 0.593548 | 16.1 | 21.2 |
| 80 | 67.5 | 78.5 | 97.5 | 54.1 | 1.05 | 29.95 | 6.663667 | 8.483333 | 4.47843 | 536.1635 | 4.8825 | 2.41475 | 53 | 21.25 | 36 | 0.590278 | 31.95 | 40.83828 |
| 81 | 70.5 | 80 | 96.75 | 53.825 | 2 | 30.15 | 6.197417 | 8.066667 | 3.653081 | 523.523 | 4.60375 | 2.34575 | 51.25 | 22.75 | 35.5 | 0.640845 | 31.075 | 38.48707 |
| 82 | 67.25 | 78.5 | 96 | 48.85 | 0.55 | 19.1 | 6.598 | 8.05 | 4.033613 | 529.7723 | 4.63325 | 2.38775 | 48 | 24.5 | 34.5 | 0.710145 | 29 | 37.91667 |
| 83 | 70.5 | 80 | 98.5 | 43.575 | 2.1 | 22.65 | 6.5425 | 8.533333 | 3.626052 | 510.7648 | 4.852 | 2.44025 | 50 | 23.5 | 35.75 | 0.657343 | 28.625 | 38.6325 |
| **Table S1.** Continued. | | | | | | | | | | | | | | | | | | |
| Genotype number | DF | DC | DM | PH (cm) | NB | NC | CD (mm) | NSC | TSW (g) | SY (kg/ha) | SL (mm) | SW (mm) | L* | a* | b* | a/b | OIL(%) | PRO(%) |
| 84 | 75.5 | 82 | 102 | 57.15 | 1.65 | 19.15 | 6.118333 | 7.75 | 3.859663 | 530.1598 | 4.7405 | 2.482 | 50.25 | 23.5 | 35.75 | 0.657343 | 31.275 | 38.375 |
| 85 | 72 | 80 | 96.75 | 49.7 | 1.65 | 37.4 | 6.449167 | 8.916667 | 3.677977 | 529.2035 | 4.55375 | 2.359 | 58 | 15.5 | 43 | 0.360465 | 30.5 | 40.51792 |
| 86 | 63 | 75.25 | 96 | 34.6 | 0.65 | 31.25 | 6.168667 | 9.133333 | 3.815355 | 824.12 | 4.744 | 2.3465 | 57.25 | 14.5 | 42.75 | 0.339181 | 30.125 | 32.58588 |
| 87 | 65.5 | 77 | 96 | 64.65 | 2.1 | 33.1 | 6.336167 | 9.25 | 3.104211 | 527.6938 | 4.46375 | 2.357 | 49.5 | 25 | 36.25 | 0.689655 | 28.375 | 38.53273 |
| 88 | 66.25 | 77 | 96 | 54 | 2.55 | 42.4 | 6.978167 | 8.516667 | 4.574969 | 527.422 | 4.8585 | 2.488 | 50 | 23 | 37 | 0.621622 | 31.7 | 39.16418 |
| 89 | 72.75 | 80 | 97.5 | 53.65 | 2.75 | 38.15 | 6.211167 | 8.3 | 4.06385 | 528.2355 | 4.467 | 2.3415 | 48.5 | 24.75 | 34.5 | 0.717391 | 31.1 | 37.68534 |
| 90 | 66.5 | 77 | 102 | 31.825 | 2.65 | 29.3 | 6.844667 | 8.2 | 4.312433 | 148.45 | 4.74625 | 2.5265 | 46.25 | 25.25 | 32.75 | 0.770992 | 31.65 | 36.25855 |
| 91 | 67.25 | 77 | 96.75 | 56 | 1.7 | 24.35 | 6.688667 | 8.516667 | 4.274892 | 439.6345 | 4.7685 | 2.47325 | 51 | 22.75 | 34.5 | 0.65942 | 31.875 | 37.90776 |
| 92 | 70.5 | 80 | 98.5 | 49.35 | 1.55 | 35.6 | 6.681 | 8.783333 | 4.164815 | 516.9748 | 4.73875 | 2.43375 | 50.25 | 24 | 36.5 | 0.657534 | 24.8 | 38.3565 |
| 93 | 67.5 | 78.25 | 96.75 | 61.1 | 0.5 | 24.05 | 6.353333 | 8.65 | 3.307799 | 526.7313 | 4.36225 | 2.2665 | 46.75 | 25.5 | 32.75 | 0.778626 | 14.95 | 20.04167 |
| 94 | 72.75 | 80 | 98.5 | 50.9 | 2.75 | 31 | 6.792333 | 8.383333 | 3.50794 | 517.4478 | 4.568 | 2.4845 | 50 | 23.5 | 38 | 0.618421 | 27.65 | 33.06875 |
| 95 | 64.75 | 77 | 98.5 | 29.35 | 2.4 | 20.95 | 6.150167 | 9.033333 | 4.925114 | 1067.54 | 4.811 | 2.4785 | 49 | 24.75 | 34.25 | 0.722628 | 32.775 | 40.00884 |
| 96 | 69.75 | 80 | 97.5 | 59.775 | 1 | 19.05 | 6.234667 | 8.716667 | 2.924935 | 521.5538 | 4.494 | 2.28325 | 48.25 | 25.75 | 33 | 0.780303 | 27.2 | 38.45083 |
| 97 | 69.75 | 77 | 97 | 59.325 | 2.8 | 41.35 | 6.767667 | 8.266667 | 4.489389 | 525.2445 | 4.946 | 2.48975 | 54.25 | 20.25 | 38.25 | 0.529412 | 30.625 | 40.39441 |
| 98 | 66.25 | 77 | 96 | 60.425 | 2.05 | 29.4 | 6.572833 | 8.066667 | 3.863281 | 538.8388 | 4.4935 | 2.3205 | 49.5 | 23.5 | 36.5 | 0.643836 | 29.9 | 39.88609 |
| 99 | 67.5 | 80.5 | 101 | 36.375 | 2.2 | 23.85 | 6.3315 | 8.416667 | 3.99021 | 549.1908 | 4.673 | 2.391 | 47.5 | 25 | 32.25 | 0.775194 | 29.425 | 34.65613 |
| 100 | 72 | 80.5 | 96 | 59.1 | 1.75 | 26.15 | 5.963667 | 8.9 | 2.941095 | 433.3588 | 4.42275 | 2.273 | 49.25 | 24.5 | 32.75 | 0.748092 | 26.475 | 40.04041 |
| 101 | 72 | 80 | 97 | 60.8 | 2.45 | 28.95 | 6.1895 | 9.25 | 3.189664 | 520.423 | 4.4295 | 2.27675 | 52.25 | 22.25 | 37.25 | 0.597315 | 29.125 | 38.21644 |
| 102 | 78.75 | 80 | 96.75 | 61.35 | 1.3 | 40.85 | 6.4115 | 7.75 | 3.029204 | 409.216 | 4.4025 | 2.29 | 46 | 13 | 35 | 0.371429 | 26.11 | 34.11 |
| 103 | 73.5 | 80 | 96.75 | 60.575 | 1.55 | 29.95 | 6.143 | 8.733333 | 3.408105 | 510.6698 | 4.7025 | 2.40325 | 52 | 25.25 | 34 | 0.742647 | 28.4 | 38.66637 |
| 104 | 73.5 | 80 | 97.75 | 43.25 | 2.8 | 41.65 | 6.418667 | 6.733333 | 3.727201 | 522.8993 | 4.7215 | 2.3605 | 60.75 | 15.25 | 49 | 0.311224 | 31.65 | 37.48361 |
| 105 | 62 | 75.25 | 96 | 37.025 | 1.9 | 23.3 | 7.3835 | 9.3 | 6.549802 | 433.9158 | 5.05125 | 2.60675 | 47.5 | 26 | 32 | 0.8125 | 34.1 | 38.61055 |
| 106 | 69.75 | 77 | 96 | 60.875 | 1 | 26.2 | 6.102833 | 9.366667 | 3.942839 | 546.6943 | 4.44425 | 2.326 | 48.5 | 28 | 32.25 | 0.868217 | 33.075 | 41.57083 |
| 107 | 79 | 87.75 | 106.5 | 38.275 | 2.4 | 16.95 | 5.939833 | 7.854167 | 4.519344 | 435.3218 | 4.52675 | 2.379 | 51.75 | 23.5 | 39.25 | 0.598726 | 14.05 | 18.25327 |
| 108 | 80.25 | 87.75 | 106.5 | 43.775 | 3.9 | 42 | 6.0285 | 8.3 | 3.595843 | 531.2775 | 4.66175 | 2.3865 | 52.75 | 23.5 | 36 | 0.652778 | 29.15 | 40.47917 |
| 109 | 73.25 | 84 | 103 | 45.1 | 3.2 | 35.15 | 6.23 | 8.633333 | 3.976652 | 541.6415 | 4.58825 | 2.4085 | 51.5 | 24 | 35.75 | 0.671329 | 31.625 | 38.92167 |
| 110 | 65.5 | 78.5 | 96 | 53.525 | 2.05 | 40.55 | 6.1725 | 9.2 | 3.434905 | 445.3098 | 4.55325 | 2.32525 | 49 | 25.25 | 33.25 | 0.759398 | 30.425 | 38.13582 |
| 111 | 65.5 | 77 | 96 | 48.5 | 1 | 23.25 | 6.458833 | 8.833333 | 3.565129 | 529.9725 | 4.53675 | 2.3385 | 49.75 | 26.5 | 35.5 | 0.746479 | 28.8 | 38.225 |
| 112 | 68.75 | 77 | 98.5 | 54.175 | 1.7 | 23.65 | 6.7195 | 8.45 | 4.355586 | 542.8695 | 4.77775 | 2.49475 | 47.5 | 25.25 | 32 | 0.789063 | 33.62884 | 39.515 |
| 113 | 66.5 | 77 | 96 | 51.2 | 2.6 | 23.05 | 6.836667 | 8.383333 | 4.276038 | 549.424 | 4.71025 | 2.501 | 47.75 | 27 | 32.5 | 0.830769 | 32.725 | 36.53712 |
| 114 | 66.25 | 77 | 96 | 49.375 | 1.5 | 25.2 | 6.137167 | 9.05 | 3.844921 | 526.9893 | 4.56 | 2.36925 | 49.25 | 24.75 | 34 | 0.727941 | 27.95 | 38.51 |
| 115 | 72 | 80 | 96.75 | 55.35 | 1.35 | 25.5 | 6.339833 | 7.6 | 3.20774 | 510.6365 | 4.6645 | 2.3555 | 63.75 | 14 | 43 | 0.325581 | 30.075 | 36.4375 |
| 116 | 72.75 | 80 | 97.75 | 50.85 | 2.45 | 22.1 | 6.557333 | 8.183333 | 3.953741 | 511.8445 | 4.8005 | 2.41275 | 52.25 | 23.25 | 38 | 0.611842 | 28.475 | 36.35417 |
| 117 | 69 | 80 | 105.5 | 38.575 | 2.8 | 37.75 | 6.196 | 8.333333 | 4.006294 | 920.32 | 4.6885 | 2.41425 | 50.5 | 23.25 | 37.75 | 0.615894 | 30.625 | 36.525 |
| 118 | 72.75 | 80 | 97.5 | 37.225 | 1.4 | 29.1 | 6.102333 | 8.35 | 3.154099 | 511.992 | 4.38275 | 2.3095 | 46.25 | 28 | 34.5 | 0.811594 | 29.375 | 39.2375 |
| 119 | 69.5 | 77 | 96 | 55.725 | 1.2 | 24.1 | 5.903667 | 8.85 | 2.731635 | 418.4775 | 4.30275 | 2.242 | 48 | 27 | 32.5 | 0.830769 | 28.175 | 36.86667 |
| 120 | 72 | 77 | 96 | 58.025 | 0.55 | 24.15 | 5.969625 | 8.966667 | 2.578855 | 514.4438 | 4.3275 | 2.24575 | 49 | 26.5 | 33.5 | 0.791045 | 24.63898 | 36.41208 |
| 121 | 71.75 | 78.5 | 96.75 | 64.425 | 2.15 | 48.9 | 5.8755 | 6.25 | 2.459755 | 513.8728 | 4.53225 | 2.31175 | 58.75 | 10.75 | 42 | 0.255952 | 29.61364 | 32.23958 |
| 122 | 67.5 | 77 | 97.5 | 46.125 | 1.9 | 37.45 | 6.298333 | 8.3 | 4.198661 | 544.9695 | 4.6915 | 2.419 | 47.5 | 26 | 33.5 | 0.776119 | 31.8 | 35.14583 |
| 123 | 67.5 | 77 | 97.75 | 45.275 | 2.25 | 40.3 | 6.304667 | 8.616667 | 3.903281 | 531.7638 | 4.65975 | 2.4345 | 49.75 | 25.75 | 35 | 0.735714 | 33.175 | 33.09417 |
| 124 | 66.25 | 77 | 96 | 75.075 | 2 | 27 | 6.415167 | 9.05 | 3.418218 | 529.7548 | 4.49475 | 2.34175 | 51.75 | 22.5 | 36.5 | 0.616438 | 28.25 | 40.64689 |
| 125 | 73.5 | 80 | 96.75 | 62.725 | 2.55 | 31.8 | 6.4835 | 8.2 | 3.415118 | 414.5708 | 4.53025 | 2.38075 | 60.25 | 14.5 | 46.75 | 0.31016 | 30.675 | 36.19833 |
| **Table S1.** Continued. | | | | | | | | | | | | | | | | | | |
| Genotype number | DF | DC | DM | PH (cm) | NB | NC | CD (mm) | NSC | TSW (g) | SY (kg/ha) | SL (mm) | SW (mm) | L* | a* | b* | a/b | OIL(%) | PRO(%) |
| 126 | 73.75 | 80 | 97.5 | 59.475 | 1.95 | 21.2 | 6.303833 | 8.516667 | 3.564154 | 523.3765 | 4.575 | 2.3965 | 50.25 | 23.75 | 36 | 0.659722 | 31.575 | 37.75 |
| 127 | 67.5 | 80.5 | 100.25 | 44.8 | 2.2 | 28.15 | 6.348667 | 8.483333 | 4.427197 | 796.56 | 4.47825 | 2.377 | 49.75 | 23 | 33.5 | 0.686567 | 29.3 | 38.35833 |
| 128 | 70.5 | 78.5 | 96.75 | 49.675 | 1.75 | 23.95 | 5.691788 | 8.657576 | 3.140603 | 521.4163 | 4.493 | 2.354 | 53.25 | 21 | 39.5 | 0.531646 | 30.65 | 36.30417 |
| 129 | 72 | 78.5 | 96.75 | 56.25 | 1.9 | 44.2 | 6.4155 | 7.65 | 4.005 | 416.789 | 4.6425 | 2.38775 | 51.75 | 22.5 | 35.5 | 0.633803 | 29.625 | 35.74583 |
| 130 | 69 | 80 | 96 | 42.125 | 2.2 | 33.6 | 6.596 | 8.216667 | 4.300916 | 550.56 | 4.8875 | 2.4175 | 50.5 | 23.75 | 37.5 | 0.633333 | 27.925 | 38.7125 |
| 131 | 67.25 | 77 | 96 | 47.475 | 1.7 | 25.85 | 6.811333 | 8.6 | 3.781997 | 514.6635 | 4.62975 | 2.3925 | 48.5 | 27.5 | 35.5 | 0.774648 | 27.875 | 35.52917 |
| 132 | 79 | 87.75 | 106.5 | 43.525 | 3 | 26.4 | 6.144333 | 8.95 | 3.582681 | 552.6945 | 4.5835 | 2.4015 | 50 | 23.75 | 36 | 0.659722 | 37.225 | 40.0125 |
| 133 | 63.75 | 77 | 96.75 | 44.1 | 2.5 | 42.7 | 6.113833 | 9.4 | 3.360542 | 539.4648 | 4.49175 | 2.29375 | 49.25 | 24.25 | 32.25 | 0.751938 | 31.15 | 38.21667 |
| 134 | 72.75 | 82 | 96 | 61.75 | 1.55 | 29.05 | 5.905833 | 9.25 | 2.999328 | 525.041 | 4.38825 | 2.27 | 46 | 24.5 | 29.5 | 0.830508 | 29.05 | 39.49167 |
| 135 | 69 | 82.25 | 104.5 | 30.1 | 2.35 | 20.9 | 6.511167 | 8.983333 | 5.058917 | 527.11 | 4.7995 | 2.51475 | 51.75 | 23.75 | 36 | 0.659722 | 31.8 | 40.4 |
| 136 | 67.25 | 80.75 | 104.5 | 30.2 | 2.1 | 18.45 | 6.405333 | 8.8 | 4.805026 | 568.43 | 4.7355 | 2.47225 | 50.75 | 23.25 | 37 | 0.628378 | 34.825 | 38.05417 |
| 137 | 71 | 77 | 96 | 59.95 | 1.6 | 26.2 | 6.506 | 8.6 | 3.571811 | 520.3248 | 4.559 | 2.39375 | 50.25 | 24.25 | 36.75 | 0.659864 | 29.0141 | 38.69167 |
| 138 | 68.75 | 77 | 96.75 | 62.75 | 1.55 | 20.05 | 6.421833 | 7.691667 | 3.108392 | 521.232 | 4.576 | 2.424 | 49.75 | 24.25 | 34.5 | 0.702899 | 29.56245 | 20.14167 |
| 139 | 69.75 | 77 | 96 | 65.55 | 0.65 | 16.4 | 6.384333 | 8.75 | 3.356456 | 303.9278 | 4.5825 | 2.3735 | 50.25 | 25.5 | 35.75 | 0.713287 | 25.5 | 39.3925 |
| 140 | 72 | 77 | 96 | 66.225 | 1.05 | 17.1 | 6.24475 | 8.216667 | 3.089179 | 306.4475 | 4.38 | 2.3005 | 52.25 | 24.5 | 37.75 | 0.649007 | 29.81303 | 18.16667 |
| 141 | 73.75 | 78.5 | 96.75 | 58.125 | 2.2 | 30.65 | 6.052167 | 6.883333 | 2.802726 | 305.7473 | 4.6075 | 2.35875 | 39.5 | 17.25 | 29.25 | 0.589744 | 21.11 | 10.3 |
| 142 | 70 | 77 | 96 | 61.1 | 0.95 | 16.05 | 6.398409 | 7.654167 | 3.549874 | 511.9605 | 4.605 | 2.435 | 50 | 26.25 | 35 | 0.75 | 30.225 | 18.15833 |
| 143 | 73.75 | 77 | 96 | 43.15417 | 1.975 | 30.6 | 6.982028 | 8.575 | 3.762751 | 306.3805 | 4.6495 | 2.4475 | 53.75 | 23 | 39.25 | 0.585987 | 29.85 | 17.32 |
| 144 | 73.75 | 77 | 96.75 | 48 | 3.15 | 22.9125 | 7.3585 | 8.125 | 4.697216 | 307.8575 | 5.071 | 2.551 | 52.5 | 25 | 38.25 | 0.653595 | 29.32 | 26.33 |
| DF days to flowering, DC days to capsule formation, DM days to maturity, PH plant height, NB number of branches, NC number of capsule, NSC number of seed per capsule, CD capsule diameter, TSW thousand seed weight, SY seed yield, SL seed length, SW seed width, L*, a*, b*, and a/b color parameters, OIL oil content, PRO protein content. | | | | | | | | | | | | | | | | | | |
